# Aerobic and Resistance Exercise Modalities Ameliorate Hippocampal Plasticity and Neurocognitive Changes Induced by Receiving Nandrolone Decanoate in Rats (Physiological, Biochemical, and Histological Study)

**DOI:** 10.3390/biology15151274

**Published:** 2026-08-03

**Authors:** Wesam M. R. Ashour, Abeer A. Khalefa, Lamiaa Hassan Ibrahim Ahmed, Enas N. Morgan, Saleh M. Aloraini, Ayman M. Mousa, Rasha A. Elmansy, Hadeel Ayman Elsherbini Eid

**Affiliations:** 1 Department of Physiology, College of Medicine, Zagazig University, Zagazig 44511, Egypt; wesam_ashour@yahoo.com (W.M.R.A.); abeerbiomy@zu.edu.eg (A.A.K.); hassanlamia242@gmail.com (L.H.I.A.); h.adeel.83@hotmail.com (H.A.E.E.); 2 Department of Physical Therapy, College of Applied Medical Sciences, Qassim University, Buraydah 51452, Saudi Arabia; imrjan@qu.edu.sa (E.N.M.); saloraini@qu.edu.sa (S.M.A.); 3 Department of Basic Health Sciences, College of Applied Medical Sciences, Qassim University, Buraydah 51452, Saudi Arabia; 4 Department of Histology and Cell Biology, Faculty of Medicine, Benha University, Benha 13518, Egypt; 5 Department of Anatomy and Histology, College of Medicine, Qassim University, Buraydah 51452, Saudi Arabia; r.elmansi@qu.edu.sa

**Keywords:** hippocampus, anabolic steroids, oxidative stress, exercise, cognition

## Abstract

Anabolic-androgenic steroids (AAS), such as ND, are commonly used to enhance physical performance and muscle mass. However, its abuse has been associated with numerous adverse health effects, particularly on the hippocampus, affecting learning, working memory, and cognitive functions. The current study aims to investigate the potential effects of ND, aerobic exercise, resistance exercise, and the combination of ND with aerobic or resistance exercise on memory and cognitive functions. Moreover, the study aims to explore the possible underlying mechanisms of changes in memory and cognitive function. Adult male rats were divided into four groups: control (GI), only ND-treated (GII), only exercise (GIIIa & GIIIb), and combined exercise + ND-treated (GIVa & GIVb). Exercise included aerobic treadmill training and ladder-climbing modalities for 8 weeks, either alone or with ND. Cognitive function was evaluated using the Modified Barnes Maze and T-maze tests. Hippocampal integrity was assessed through oxidative stress markers, hippocampal brain-derived neurotrophic factor (BDNF) levels, hippocampal nerve growth factor (NGF) expression, and histological examination. ND significantly impaired spatial learning and memory, increased oxidative stress, lowered hippocampal BDNF levels, and reduced NGF expression in the hippocampus. Exercise markedly attenuated these harmful effects. Aerobic exercise gave the most pronounced improvements. It restored cognitive function, enhanced antioxidant defenses, increased hippocampal BDNF levels, increased hippocampal NGF expression, and preserved hippocampal structure. Resistance training also had neuroprotective effects, but to a lesser extent than aerobic exercise. In brief, both aerobic and resistance exercise have been shown to exert protective effects against ND-induced neurotoxicity by enhancing antioxidant defenses and promoting neuronal integrity. The monitored neuroprotective benefits suggest that physical exercise could serve as a possible non-pharmacological approach to prevent or decrease cognitive impairments associated with anabolic steroid abuse.

## Simple Summary

The current study tested whether nandrolone decanoate (ND), a steroid used to build muscle, harms memory and brain function, and examined whether aerobic or resistance exercise could protect against these harmful effects in rats. The rats were divided into control, steroid-only, exercise-only, and combined treatment groups. Their learning and memory were measured with maze tests. The researchers also checked the hippocampus for oxidative stress, BDNF, NGF, and tissue damage. ND worsened learning and memory, increased oxidative stress, and reduced important brain-support factors. Exercise helped reduce these negative changes and protect the hippocampus. Aerobic exercise showed the strongest benefit, while resistance exercise also helped, albeit to a lesser extent. Overall, the study suggests that regular exercise may help protect the brain from steroid-related damage.

## 1. Introduction

The hippocampus is a brain region primarily responsible for memory building. In rats, it consists of several subregions, including the dentate gyrus and the CA1, CA2, CA3, and CA4 subfields. The dentate gyrus contains a layer of densely packed granule cells, an adjacent subgranular zone, and an additional molecular layer. Granule cells play a vital role in the formation of new memories and are characterized by their distinct morphology and prominent granules [1].

Anabolic-androgenic steroids (AAS) abuse is a public health problem for both athletes and non-athletes, including adolescents and young adults [2]. Overall, AAS prevalence is approximately 4%, reaching 16.8% among bodybuilders and 4.4% among athletes and recreational gym users [3]. Globally, prevalence rates are notable among 20% of US athletes [4], 22% of UAE gym users [5], 13% of Iranian youth bodybuilders [6], and 29.3% of Riyadh gym users [7]. In Egypt, AAS use was reported among 32% of supplement users and 57% of athletes [8]. AAS are synthetic derivatives of testosterone [9]. Nandrolone decanoate (ND) is frequently abused for its strong anabolic effects [10]. Clinically, AAS are used to treat osteoporosis and muscle-wasting diseases [11]. However, at high doses, AAS can alter central nervous system structure and function [12], particularly affecting the hippocampus, a key region for cognition and behavior [13].

Experimental studies indicate that administration of AAS can induce anxiety, depression, memory deficits, and structural changes in critical brain regions, including the hippocampus, amygdala, and prefrontal cortex [14]. In humans, prolonged use of these substances is associated with increased amygdala volume, cortical thinning, and reduced grey matter [14,15]. ND administration can also increase the levels of proinflammatory cytokines, such as TNF-α and IL-1β [16].

Physical exercise confers significant cognitive and mental health benefits. It reduces the risk of depression and improves the overall brain health [17]. Exercise promotes neuroplasticity by increasing brain-derived neurotrophic factor (BDNF), improving memory, and stimulating neurogenesis [18]. Aerobic training has been associated with increased hippocampal volume [19]. Appropriately timed exercise may also cause protection against inflammation associated with certain diseases [20]. The effects of these benefits depend on the type, intensity, and duration of exercise [21]. According to World Health Organization guidelines, any level of physical activity is preferable to inactivity [22]. Therefore, the current study aimed to investigate the effects of different exercise modalities (moderate aerobic and resistance exercise training) on cognitive functions, oxidative stress markers, hippocampal BDNF levels and NGF expression, and hippocampal morphological changes in rats, and to clarify the underlying mechanisms with and without chronic supraphysiological ND administration.

## 2. Materials and Methods

### 2.1. Drugs and Chemicals

ND (Deca-Durabolin 200 mg/mL in arachis oil), Alpha MD (Lynnwood, USA). SOD (CUSABIO, CSB-E08555r), MDA (MYBIOSOURCE, MBS268427), and BDNF ELISA (CUSABIO, CSB-E04504r) kits were used. Routine histology reagents (10% formalin and H&E stains) were used. The recombinant nerve growth factor (NGF) protein was obtained from Abcam (Cambridge, UK; ab269181).

### 2.2. Study Design

Thirty-six adult male albino rats (200–250 g, 9 weeks old) were obtained from the Laboratory Animal House, Faculty of Veterinary Medicine, Zagazig University. Animals were housed in standard steel cages under monitored temperature (24 ± 2 °C) and a natural light/dark cycle. Rats were acclimatized for 2 weeks before starting the experiment [23]. All procedures followed the Guidelines for the Care and Use of Laboratory Animals (1996) and were approved by the Institutional Animal Care and Use Committee of Zagazig University, ZU-IACUC (No. ZU-IACUC/3/F/281/2026). Rats were randomly assigned to four experimental groups using a computer-generated randomization method to minimize sampling bias. Rats were given identification numbers from 1 to 36, and a computer-generated random sequence was created using the random number generation function in Microsoft Excel. Animals were then allocated to the six experimental groups in the order generated. The sample size was chosen based on previous comparable experimental studies and in accordance with ethical principles to minimize animal use. The control group (GI, *n* = 6) received 0.15 mL of a vehicle (Arachis oil, IM) 5 times/week for 8 weeks. The ND group (GII, *n* = 6) received 15 mg of ND/kg/day of ND IM, 5 times/week for 8 weeks. This dose regimen represents a supraphysiological regimen that mimics chronic AAS abuse [24] and can induce measurable neurobehavioral, oxidative, and histopathological alterations. The exercise groups were subdivided into two equal groups: aerobic treadmill training (GIIIa, *n* = 6) and anaerobic ladder-climbing training (GIIIb, *n* = 6). The exercise + ND groups were subdivided into two equal groups: aerobic exercise + ND (GIVa *n* = 6) and ladder-climbing resistance training + ND (GIVb *n* = 6). Behavioral assessments were conducted by investigators blinded to group allocation whenever feasible. Biochemical, histopathological, immunohistochemical evaluation, and morphometric measurements were performed by independent investigators blinded to treatment conditions. The exclusion criteria consisted of rats weighing less than 200 g, severe illness, accidental injury, or inability to complete the assigned exercise protocol.

### 2.3. Exercise Training Protocols

#### 2.3.1. Aerobic Training Rats

Subgroups GIIIa and GIVa underwent a one-week habituation period to become familiar with the treadmill environment. During this period, they were gently trained to run at a low speed (5 m/min for 10 min), using minimal tactile stimulation (a gentle push with a small stick) as needed to encourage forward movement. Then rats ran on a motorized treadmill 5 times/week for 8 weeks at progressively increasing intensity (10–20 m/min; 25–30 min/session) without electrical shock [25,26].

#### 2.3.2. Resistance (Anaerobic) Training Rats

Subgroups GIIIb and GIVb were first habituated to the resistance-training apparatus for 1 week, which consisted of a 1 m ladder inclined at 80°. A dark, enclosed box was placed at the top to encourage climbing and to provide a safe resting area between trials [26]. After habituation, they completed 8 climbs/session, 5 times/week for 8 weeks, with progressively increasing tail-attached loads [24,26].

### 2.4. Behavioral Assessment

#### 2.4.1. Barnes Maze Test

Spatial learning and memory were evaluated following standard habituation (Day 0), acquisition (Days 1–3), and probe trials (Days 4 and 9; PT1 and PT2, respectively). Each rat underwent two sessions. There was a 4-day rest (Days 5, 6, 7, and 8). Escape latency and error frequency were recorded [27].

#### 2.4.2. T-Maze Spontaneous Alternation Test

Working memory was evaluated over two consecutive days following established spontaneous alternation methods [28]. A percentage of correct choice (alternation) per animal was calculated as follows: Number of correct choices (Alternations)/Total possible alternations × 100.

#### 2.4.3. Rotarod Test

Motor coordination and endurance were assessed using an accelerating rotarod (5 –40 rpm). Latency to fall was recorded [29].

### 2.5. Biochemical Assays

Twenty-four hours after behavioral testing, overnight-fasted rats were anesthetized, and blood was collected from the orbital sinus to measure serum levels of superoxide dismutase (SOD) and malondialdehyde (MDA). Serum was separated (3000 rpm, 15 min) and stored at −20 °C [30]. Serum SOD activity was measured using a sandwich ELISA kit (Cat. No: MBS040427) [31]. Serum MDA was determined by competitive ELISA [32]. Each rat’s brain was bisected into two hemispheres; one hemisphere was stored at −80 °C to obtain hippocampal BDNF from hippocampal brain tissue homogenate. The second hemisphere was fixed in 10% formalin, embedded in paraffin blocks, and sectioned at 4 µm thickness. Hippocampal BDNF was quantified in brain homogenate using a quantitative sandwich ELISA [33].

### 2.6. Histopathology and Immunohistochemistry Study

Alternate sections from each paraffin-embedded block were stained with H&E, and the other sections were immunostained for NGF [34]. The DAKO EnVision+ system was used for detection, and Diaminobenzidine (DAB) was used as a chromogen to visualize immunoreactivity after antigen retrieval and incubation with a monoclonal primary antibody, followed by counterstaining with Mayer’s hematoxylin [35].

### 2.7. Quantitative Morphometric Analysis

Quantitative morphometric analysis was performed on ten non-overlapping fields per section at ×400 magnification using a light microscope with a digital camera (Olympus CX23, Tokyo, Japan). H&E–stained sections were used to calculate the thickness of GCL in the DG, and the immunostained sections were analyzed for the number of neurons showing NGF immunoreactivity using ImageJ (version 22).

### 2.8. Statistical Analysis

Researchers of the current study collected, tabulated, and statistically analyzed all data using IBM SPSS Statistics for Windows, Version 23.0 (IBM Corp., Armonk, NY, USA, 2015). They used the Shapiro–Wilk test to assess the normality of quantitative variables and reported descriptive statistics as mean ± standard deviation and ranges. To compare more than two normally distributed groups, they used ANOVA. If the F-test was significant, they used the LSD test for each pair to identify significant groups. All tests considered a two-sided *p*-value < 0.05 statistically significant, and a *p*-value ≥ 0.05 as statistically non-significant. Effect sizes were calculated using eta-squared (η^2^).

### 2.9. AI-Assisted Language Revision

The authors used ChatGPT (OpenAI, San Francisco, CA, USA; version 5.5, 2025) as an AI-assisted writing tool to refine the manuscript’s English. The authors carefully reviewed and edited the modified content and take full responsibility for the final content and integrity of the manuscript, including the accuracy of the data, interpretations, and conclusions.

## 3. Results

### 3.1. Behavioral Assessment Results

#### 3.1.1. Modified Barnes Maze Test

Table 1 shows the Escape Latency Across Acquisition Trials (AT1–AT6). Across all acquisition trials, chronic ND administration (GII) induced a severe and persistent impairment in spatial learning, as evidenced by a significant prolongation in escape latency compared to GI (*p* < 0.001 for all trials). This impairment was strong and did not attenuate with repeated training, indicating that ND induced a marked impairment in hippocampal-dependent learning. Contrarily, both aerobic (GIIIa) and anaerobic (GIIIb) exercise markedly improved learning performance. Exercise significantly shortens escape latencies compared with ND-treated rats across all trials (*p* < 0.001). In the early learning phase (AT1–AT2), exercised rats performed better than controls (*p* < 0.05), suggesting that exercise facilitated acquisition. No significant difference was detected between the effects of aerobic and anaerobic exercises (*p* > 0.05). In the combined treatment groups (GIVa and GIVb), aerobic and ladder-climbing resistance training partially improved ND-induced impairment. Rats receiving ND combined with exercise (GIVa and GIVb) showed a significant decrease in escape latency compared with ND alone (*p* < 0.001). However, their performance was less than that of the exercise-only groups (GIIIa and GIIIb) (*p* < 0.001 in most trials). Aerobic training (GIVa) generally exerted a greater protective effect than ladder-climbing resistance training (GIVb), as evidenced by longer latencies across several trials. In all Barnes maze escape latency trials (AT1–AT6), ND treatment and exercise modality consistently yielded large effect sizes (η^2^ = 0.972–0.982), indicating potent, biologically significant, and consistent effects on spatial learning acquisition and cognitive performance.

Table 2 shows the Number of Errors Across Acquisition Trials (AT1–AT6). The error analysis results aligned with the latency results. GII showed the highest error counts across all trials, far outmatching those of controls (*p* < 0.001), reflecting an impaired spatial search strategy and reduced learning aptitude. Exercise-only groups (GIIIa and GIIIb) showed a markedly reduced number of errors relative to both GI and GII across nearly all trials (*p* < 0.05 vs. GI; *p* < 0.001 vs. GII). Aerobic and ladder-climbing resistance training did not differ significantly (*p* > 0.05). In the combined groups, both GIVa and GIVb demonstrated intermediate performance. While errors were significantly fewer than in GII rats (*p* < 0.001), they remained elevated compared with exercise-only subgroups (GIIIa and GIIIb) (*p* < 0.001). GIVb made more errors than GIVa, indicating higher neuroprotection with aerobic training when combined with ND than anaerobic resistance training. In all Barnes maze tests, the number of errors across trials (AT1–AT6), ND treatment, and exercise modality showed consistently large effect sizes (η^2^ = 0.900–0.987), indicating robust, biologically meaningful, and consistent effects on spatial learning acquisition and cognitive performance.

Table 3 shows the Probe trials (PT1 & PT2), which measure spatial reference memory and retention of the learned escape location, demonstrated a compatible pattern across all experimental groups. ND administration produced a considerable impairment in memory performance, as evidenced by the markedly prolonged escape latencies in both PT1 and PT2 compared with controls (*p* < 0.001). This shows a significant disruption of hippocampal-dependent spatial memory recollection following chronic ND exposure. In contrast, both aerobic and ladder-climbing resistance training produced strong memory-enhancing effects. Rats in subgroups GIIIa and GIIIb showed the shortest latencies in both probe trials compared to those in GI and GII (*p* < 0.001 for all comparisons). These data suggest that regular physical exercise improves memory quality and recovery capabilities, with significant efficacy for both aerobic and anaerobic training. When exercise was combined with ND in GIVa and GIVb, escape latencies improved compared with ND alone, indicating partial protection against ND-induced cognitive impairment. However, these ND and exercise-combined subgroups (GIVa & GIVb) showed longer latencies than the exercise-only subgroups (GIIIa and GIIIb) (*P* > 0.001 for both). Aerobic training (GIVa) is significantly higher than ladder-climbing resistance training (GIVb), indicating a greater neuroprotective effect of aerobic exercise against ND-induced neurotoxicity. In both probe trials (PT1 & PT2), ND treatment and exercise modality showed large effect sizes (η^2^ = 0.988–0.991), indicating a potent and significant effect on spatial memory retrieval and retention.

Table 4 shows the Number of Errors in Probe Trial (PT1 & PT2). Performance of rats during PT1 and PT2 was fully consistent with the latency outcomes, further confirming the effects of ND and exercise on spatial memory accuracy. Rats administered ND alone (GII) exhibited the highest number of errors in both probe trials, exceeding those of all other groups (*p* < 0.001 for all). This elevated error rate indicated a significant impairment in spatial memory recall, as reflected in a decrease in the rats’ ability to correctly identify the target hole, consistent with ND-induced hippocampal dysfunction. Conversely, both aerobic and ladder-climbing resistance training subgroups (GIIIa and GIIIb) showed the most accurate performance, achieving the fewest errors across both Probe trials (*p* < 0.001 vs. GI and GII). This improvement indicates the strong memory-enhancing effect of routine physical training and its role in improving spatial search strategies. In contrast, the combination of exercises with ND treatment in both subgroups GIVa and GIVb showed significantly fewer errors than GII, indicating partial neuroprotection conferred by exercises against ND-induced impairment. While error rates remained higher than those observed in navigational performance, showing fewer errors and better navigation accuracy during AAS exposure than GIVb. The number of errors across both probe trials (PT1 and PT2), ND exposure, and exercise modality showed large effect sizes (η^2^ = 0.982–0.989), indicating highly significant effects on spatial memory retrieval and retention.

#### 3.1.2. The Modified T-Maze Test

Table 5 shows the results of the Modified T-Maze Test, indicating significant differences in working memory performance among groups. In GII, rats showed significantly lower correct-choice percentages than in GI (*p* < 0.001). Moreover, both exercise modalities (GIIIa and GIIIb) showed significant improvements in memory accuracy (*p* < 0.001 & *p* < 0.05, respectively), with no significant difference between the two modalities (*p* > 0.05). In contrast, the combination of exercises with ND (GIVa and GIVb) partially improved performance (*p* < 0.001); however, performance remained below the levels observed in GIIIa and GIIIb (*p* < 0.001 vs. both GIIIa and GIIIb). Aerobic exercise in GIVa showed better outcomes than ladder-climbing resistance training in GIVb (*p* < 0.05).

#### 3.1.3. Rotarod Test Latency

Table 5 shows that rotarod performance indicated significant differences in motor coordination and balance among groups. ND exposure significantly lowered latency to fall in GII compared with GI (*p* < 0.001). In contrast, GIIIa and GIIIb exhibited the longest latencies, indicating superior motor performance (*p* < 0.001 vs. all other groups). Combined training and ND (GIVa and GIVb) enhanced performance relative to ND alone (GII) (*p* < 0.001 for both), but did not reach the level of exercise-only groups and remained less than GI. Also, there were insignificant differences in performance between GIVa and GIVb. ND exposure and exercise modality resulted in large effect sizes for both Modified T-maze performance (η^2^ = 0.884) and Rotarod Latency (η^2^ = 0.968). These findings demonstrated significant impacts on memory, cognitive flexibility, motor coordination, and neuromuscular performance.

### 3.2. Biochemical Assays

Table 6 shows the results of the biochemical assays: GII showed a significant reduction in Neuroplasticity Marker levels, BDNF, compared with GI (*p* < 0.05), indicating impaired neuroplasticity. Conversely, both GIIIa and GIIIb showed higher BDNF levels exceeding those of GI and GII (*p* < 0.001 for all). In contrast, GIVa and GIVb partially restored BDNF levels but remained significantly lower than exercise alone (*p* < 0.001 for all), demonstrating that training reduces, but does not fully reverse, the neurotoxic effects of ND. The oxidative stress marker, serum MDA levels, showed their highest levels in GII (*p* < 0.001 vs. all groups), indicating severe oxidative stress, while GIIIa and GIIIb showed markedly reduced MDA levels (*p* < 0.0001 vs. all groups), demonstrating the powerful antioxidant effects of both exercise modalities. The combination of exercise and ND in subgroups GIVa and GIVb showed a significant reduction in oxidative stress compared with GII; however, they still exhibited elevated oxidative stress compared with GIIIa and GIIIb (*p* < 0.001), indicating partial protection. Moreover, the ND significantly suppressed serum SOD activity in GII (*p* < 0.001), whereas exercise modalities in both GIIIa and GIIIb dramatically increased SOD values compared with all other groups, with aerobic training showing a superior antioxidant effect compared with ladder-climbing resistance training (*p* < 0.001). In contrast, combined aerobic and ladder-climbing resistance training with ND in GIVa and GIVb improved SOD levels relative to ND alone in GII (*p* < 0.001 for all), but remained significantly less than both GIIIa and GIIIb (*p* < 0.001). ND exposure and exercise modality showed large effect sizes across all biochemical parameters, including hippocampal BDNF levels, MDA, and SOD (η^2^ = 0.997, 0.992, and 0.998, respectively). These findings indicate significant effects on neuroplasticity-related processes, oxidative stress, lipid peroxidation, and antioxidant defense.

### 3.3. Histopathological Study

Figure 1 and Figure 2. The Histopathological Examination of the Dentate Gyrus in all Experimental Groups. Figure 3. The Exhibited Immunohistochemical Examination of the DG in all Experimental Groups.

Table 7 shows the results of measuring hippocampal GCL thickness, which was markedly reduced in the GII compared with all other groups (*p* < 0.05). Aerobic exercise alone (GIIIa) produced the highest mean thickness and did not differ significantly from GI, indicating preservation of hippocampal architecture. Similarly, resistance training (GIIIb) maintained thickness at levels comparable to those of GI. In contrast, combined treatment subgroups (GIVa and GIVb) exhibited partial structural recovery. GIVa and GIVb showed significantly greater thickness than GII (*p* < 0.05) but remained significantly reduced compared with exercise-only groups, with GIVb displaying the most considerable reduction among the combined groups. Moreover, GII showed a profound reduction in the number of NGF-immunostained cells compared with all other groups (*p* < 0.05). Aerobic exercise in GIIIa markedly increased neuronal count, which was significantly higher than GI, GII, and the combined treatment subgroups (GIVa and GIVb) (*p* < 0.05). Also, resistance exercise (GIIIb) significantly increased NGF expression compared with GII, to a lesser extent than aerobic training (GIVa). The combined exercise with ND (GIVa and GIVb) improved NGF expression compared with GII (*p* < 0.05); however, both subgroups remained significantly lower than GIIIa and GIIIb, indicating partial neurotrophic protection conferred by exercise against chronic ND treatment.

## 4. Discussion

In the current study, we focused on the DG because it is the hippocampal subfield most closely linked to adult neurogenesis, pattern separation, and experience-dependent plasticity, and is often the most sensitive site for detecting subtle neurocognitive changes [36]. In contrast, the CA1 and CA3 are heavily involved in downstream memory processing and can also be affected by ND, but they are less specific for neurogenesis-driven plasticity than the DG [37]. The DG region has unique anatomical, cellular, and molecular characteristics and is highly plastic, serving as a critical interface among environmental stimuli, hormonal influences, and memory-related neural remodeling [38]. Another strong reason for studying the DG is that it is the primary site of adult hippocampal neurogenesis and the major region in the adult mammalian brain where new neurons continue to be generated [39]. Also, hippocampal plasticity depends strongly on neurotrophic factors, including the major markers BDNF and NGF, and the DG is highly sensitive to changes in their levels [40].

Several limitations should be regarded when analyzing the findings of the present study. First, the inclusion of only adult male rats limits the generalizability of the results to females, particularly given potential sex-related differences in the hormonal regulation of oxidative stress responses and hippocampal neuroplasticity. The second is that the relatively small sample size may reduce statistical power to detect relations between ND exposure and exercise modality. The third is that although the study integrated behavioral, biochemical, histological, and immunohistochemical outcomes, additional molecular analyses are needed to clarify the underlying mechanisms. Finally, the findings should be interpreted with caution on the human relevance, as experimental ND dosing paradigms may not fully reflect the pattern of AAS abuse in clinical or athletic settings, and systemic toxicity due to supraphysiological doses may have contributed to some of the observed effects. However, the effect of stress due to repeated injections was neutralized by injecting the controls with a placebo. The present findings show that prolonged ND exposure induces pronounced cognitive dysfunction accompanied by redox imbalance and structural alterations within the hippocampus. ND-treated animals exhibited impaired spatial acquisition, reduced working memory, and reduced cognitive performance across behavioral paradigms, suggesting widespread disruption of hippocampal-dependent processing. Similar patterns of morphological and cognitive impairment have been described in humans exposed to supraphysiological chronic AAS abuse [41,42]. In contrast, animals that participated in regular physical activity showed a potent protective effect. Both aerobic and resistance exercises enhanced memory and cognitive performance, with aerobic exercise showing the highly detectable benefits. These results are consistent with extensive evidence indicating that regular physical training enhances hippocampal plasticity and brain perfusion, and promotes adult neurogenesis, particularly learning and memory consolidation [43,44].

A comparable study has further suggested that endurance-based exercise may result in a greater cognitive improvement than anaerobic modality, especially in domains such as spatial navigation and executive performance [45]. The greatest outcomes observed in the aerobic group, therefore, appear biologically significant and compatible with the known sensitivity of hippocampal circuits to maintained aerobic stimulation [46]. The cognitive impairment associated with ND administration may be attributed to steroid-induced alterations in hippocampal circuitry. Previous studies have shown that supraphysiological doses of AAS affect dopaminergic signaling and modify dendritic spine density within hippocampal subfields, possibly compromising the neural substrates underlying spatial and short-term memory [47]. Biochemical analyses further support ND-induced neurotoxicity, characterized by elevated MDA levels and reduced SOD activity, demonstrating a shift toward a peroxidative state. In contrast, both exercise modalities reduced lipid peroxidation and enhanced endogenous antioxidant activity. This antioxidant support is well documented in the literature, which defines regular exercise as a potent modulator of redox homeostasis [48,49]. The lowered oxidative burden in exercised groups provides a reasonable explanation for the enhanced behavioral and structural outcomes. Furthermore, a decline in hippocampal BDNF was observed in GII. This reduction in BDNF may reflect a stress-like response induced by AAS exposure, which has been linked to increased endogenous corticosterone. Elevated corticosterone is known to suppress hippocampal BDNF expression, as demonstrated under immobilization stress conditions [50]. Conversely, the exercise groups showed elevated BDNF levels, a finding supported by accumulating evidence that physical activity enhances brain functional plasticity [51]. Exercise also increases levels of growth factors beyond BDNF, including insulin-like growth factor-1 (IGF-1) [52]. Nay et al. [53] demonstrated that four weeks of treadmill training increased hepatic IGF-1 mRNA expression in rats. Moreover, resistance exercise has been associated with higher circulating IGF-1 levels, which can cross the blood–cerebrospinal fluid barrier and promote neurogenesis, particularly in the hippocampus [54]. In the present study, histopathological investigations corroborated the proposed mechanisms and revealed reduced hippocampal thickness, neuronal loss, and degenerative cytological features in GII. Exercises, particularly aerobic training, preserved tissue morphology and reduced the degenerative changes. It was consistent with prior findings that physical activity ameliorates structural deterioration and supports hippocampal integrity [24,55].

Moreover, the immunohistochemical investigation of NGF expression suggested an additional mechanism underlying the neurobiological alterations underlying these behavioral changes. ND markedly suppressed NGF immunoreactivity, consistent with diminished neurotrophic signaling, whereas both exercise paradigms restored NGF expression, with aerobic exercise producing the greatest effect. These observations are consistent with previous reports demonstrating that exercise promotes neurotrophin expression and maintains neuronal integrity, whereas AAS exposure suppresses neurotrophic pathways [56,57]. Concurrently, these findings indicate that ND abuse exerts its cognitive and structural toxicity through varied mechanisms, including oxidative stress, neurotrophic suppression, and hippocampal degeneration. Contrarily, regular physical exercise, particularly aerobic training, counteracts these harmful effects by enhancing antioxidant defenses, preserving neuronal integrity, and upregulating BDNF.

Although direct translation from animal models to humans should be approached with caution, these findings may have translational relevance for individuals who misuse AAS to enhance muscle mass, strength, or physical appearance. The observed impairments in cognitive performance, hippocampal morphology, neurotrophic markers, and oxidative balance suggest that chronic AAS exposure may adversely affect brain function beyond its well-recognized systemic effects. Significantly, the beneficial effects observed following exercise interventions support the concept that structured physical activity may represent an accessible method to attenuate some of the neurobehavioral impairment associated with long-term AAS misuse.

## 5. Conclusions

Collectively, our study demonstrated that the convergence of behavioral, biochemical, histological, and immunohistochemical findings supports the potential of exercise as an accessible, non-pharmacological approach to reduce the neurological consequences of AAS misuse. Further studies are needed to examine the effects of varying doses and durations of AAS exposure to better understand the dose–response relationship in neurocognitive and structural brain alterations. Additionally, future research on female rats is recommended to assess sex-related differences in responses to AAS and exercise on brain function and to investigate the potential synergistic benefits of combining exercise with other neuroprotective agents to antagonize AAS-induced neurotoxicity.

## Figures and Tables

**Figure 1:**
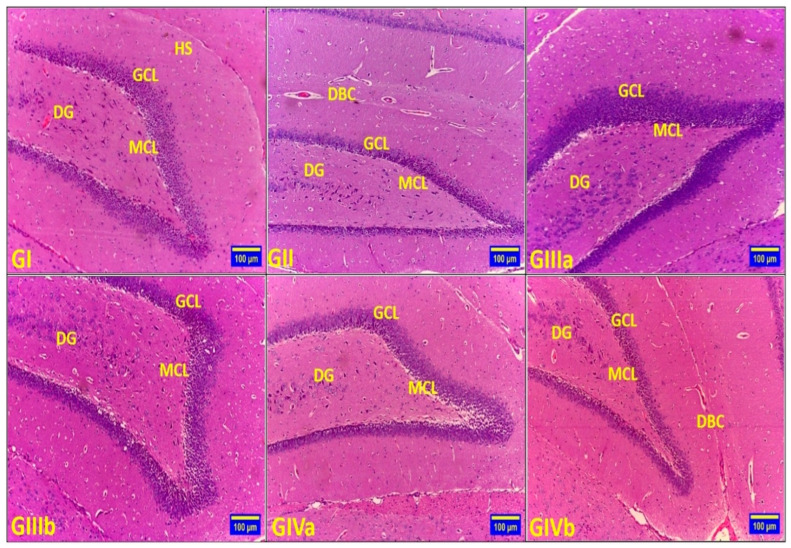
Shows representative photomicrographs from sections of the hippocampus in all groups of rats stained with H&E. The dentate gyrus (DG) below the hippocampal sulcus (HS) shows three layers [granule cell layer (GCL), subgranular zone, and molecular cell layer (MCL)]. The GCL has closely arranged neurons in the control group (GI), whereas the GCL shows a marked reduction in thickness, accompanied by numerous dilated blood capillaries (DBC), indicating neurodegenerative changes in the ND-treated group (GII). In contrast, the DG shows a marked increase in GCL thickness in the aerobic subgroup (GIIIa), with a dense neuronal arrangement, and a normal thickness in the anaerobic subgroup (GIIIb), with closely packed neurons. Also, the DG shows improvement in the aerobic+ND subgroup (GIVa), evidenced by a considerable increase in GCL thickness, and a mild improvement in the anaerobic+ND subgroup (GIVb), as indicated by an increase in GCL thickness relative to GII (H&E, ×200).

**Figure 2:**
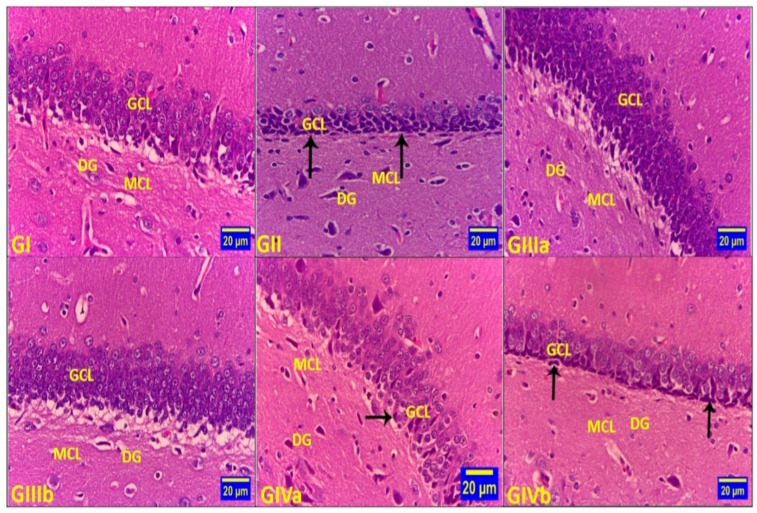
Shows representative photomicrographs from higher magnifications of the hippocampal sections in all groups of rats stained with H&E. The DG shows normal thickness of the GCL, with intact closely arranged granular neurons that exhibit normal nuclear morphology in GI, while shows a marked reduction in the thickness of the GCL, accompanied by an increased number of degenerated neurons (black arrows) at the MCL, indicating neurodegenerative changes in GII. In contrast, the DG shows a marked increase in GCL thickness, with dense, intact granular neurons in GIIIa, and a normal GCL thickness, with closely packed, intact neurons in GIIIb. Also, the DG shows a moderate improvement in GIVa, evidenced by increased GCL thickness and fewer degenerated neurons (black arrows), whereas it shows a mild improvement in GIVb, demonstrated by increased GCL thickness and fewer degenerated neurons (black arrows) compared to GII (H&E, ×400).

**Figure 3:**
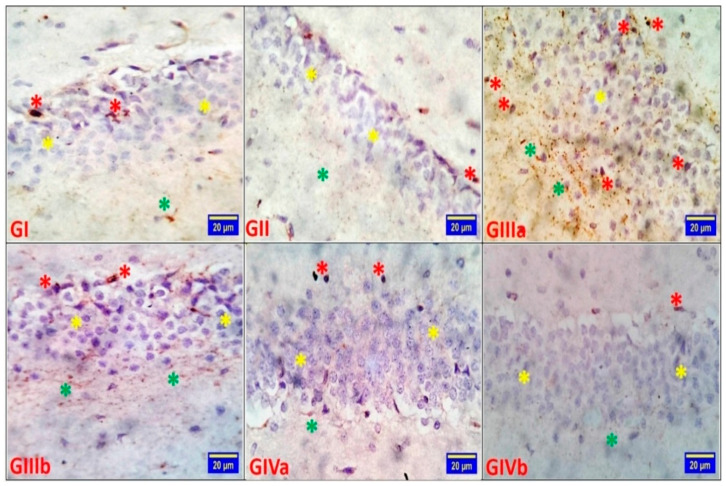
Shows representative high-magnification photomicrographs of hippocampal sections from all rat groups, immunostained for nerve growth factor (NGF). The DG shows preserved standard architecture in GI, with normal thickness of the GCL that exhibit mild cytoplasmic immunoexpression (CIE) of NGF in few scattered granule cells (red stars) and the neuropil (green stars) and negative CIE of NGF in most of the granule cells (yellow stars), whereas it shows a marked reduction in the thickness of the GCL and their neuronal density in GII, accompanied with a weak CIE of NGF in few granule cells (red stars) and negative in most of the granule cells (yellow stars), indicating neurodegenerative changes in GII. At the same time, the DG shows preserved architecture with increased cellularity in the dentate granular layer and moderate CIE of NGF in several neurons (red stars) and in the neuropil (green stars), whereas other neuronal elements appear negative (yellow stars) in GIIIa. Also, GIIIb shows a mild focal increase in GCL cellularity, with mild positive CIE of NGF in scattered neurons (red stars) and neuropil (green stars), while most neuronal elements show negative CIE of NGF (yellow stars). In contrast, the DG shows a moderate increase in GCL cellularity, with weak CIE of NGF in a few neurons (red stars) and the neuropil (green stars), while most neuronal elements remain negative (yellow stars) in GIVa. Also, the DG shows a mild focal increase in the cellularity of the dentate GCL with weak CIE of NGF in a few neurons (red stars) and the neuropil (green stars), while most of the neuronal elements remain negative (yellow stars), showing a mild improvement in GIVb compared to GII (NGF, ×400).

**Table 1:** Escape latency (sec) during Barnes maze acquisition trials (AT1–AT6) in the experimental groups.

Group	AT1	AT2	AT3	AT4	AT5	AT6
GI	52.67 ± 3.26	35.33 ± 5.06	41.17 ± 2.64	32.67 ± 2.58	37.33 ± 3.20	33.33 ± 1.97
GII	117.83 ± 2.14 *	111.0 ± 7.23 *	113.67 ± 3.61 *	115.0 ± 4.29 *	115.0 ± 4.29 *	99.83 ± 7.98 *
GIIIa	43.17 ± 1.17 *,$	24.0 ± 1.79 *,$	38.33 ± 2.87 $	28.17 ± 2.32 $	28.0 ± 2.83 *,$	19.83 ± 2.32 *,$
GIIIb	44.33 ± 1.63 *,$	25.83 ± 2.32 *,$	38.50 ± 2.66 $	30.83 ± 3.31 $	30.83 ± 3.31 *,$	22.33 ± 1.51 *,$
GIVa	69.83 ± 3.43 *,$,#,^	49.0 ± 3.89 *,$,#,^	56.67 ± 4.55 *,$,#,^	45.33 ± 3.78 *,$,#,^	44.5 ± 3.39 *,$,#,^	42.67 ± 4.59 *,$,#,^
GIVb	86.50 ± 7.56 *,$,#,^,†	56.33 ± 4.18 *,$,#,^,†	65.0 ± 7.32 *,$,#,^,†	51.67 ± 7.55 *,$,#,^	59.3 ± 7.86 *,$,#,^,†	49.5 ± 4.14 *,$,#,^,†
η^2^ (Eta-squared)	0.982	0.951	0.930	0.980	0.975	0.972

Data are expressed as mean ± SD (*n* = 6). Significance = *p* < 0.05; * significant vs. GI; $ significant vs. GII; # significant vs. GIIIa; ^ significant vs. GIIIb; † significant vs. GIVa.

**Table 2:** Number of errors during Barnes maze acquisition trials (AT1–AT6) in all experimental groups.

Group	AT1	AT2	AT3	AT4	AT5	AT6
GI	7.17 ± 0.76	5.5 ± 1.05	4.83 ± 0.75	3.67 ± 0.82	4.0 ± 0.89	3.5 ± 0.55
GII	12.83 ± 0.98 *	11.33 ± 0.82 *	11.67 ± 1.03 *	13.0 ± 0.89 *	11.0 ± 0.89 *	11.18 ± 0.98 *
GIIIa	4.33 ± 0.52 *,$	2.67 ± 0.82 *,$	2.17 ± 0.41 *,$	1.33 ± 0.52 *,$	2.17 ± 0.41 *,$	1.17 ± 0.41 *,$
GIIIb	4.83 ± 0.98 *,$	2.83 ± 0.98 *,$	2.33 ± 0.52 *,$	1.50 ± 0.55 *,$	2.50 ± 0.55 *,$	1.50 ± 0.55 *,$
GIVa	7.83 ± 0.98 $,#,^	7.50 ± 1.05 *,$,#,^	6.17 ± 1.17 *,$,#,^	5.0 ± 0.89 *,$,#,^	5.5 ± 0.55 *,$,#,^	4.33 ± 0.52 *,$,#,^
GIVb	8.50 ± 1.05 *,$,#,^	8.0 ± 1.26 *,$,#,^	7.5 ± 1.05 *,$,#,^,†	6.0 ± 0.89 *,$,#,^,†	6.5 ± 1.04 *,$,#,^,†	6.0 ± 0.89 *,$,#,^,†
η^2^ (Eta-squared)	0.925	0.900	0.954	0.981	0.964	0.987

Data are expressed as mean ± SD (*n* = 6). Significance = *p* < 0.05; * significant vs. GI; $ significant vs. GII; # significant vs. GIIIa; ^ significant vs. GIIIb; † significant vs. GIVa.

**Table 3:** Escape latency (sec) during probe trials (PT1 & PT2) in the experimental groups.

Group	PT1	PT2
GI	24.17 ± 2.04	22.83 ± 3.25
GII	116.17 ± 4.22 *	117.17 ± 3.43 *
GIIIa	17.33 ± 2.06 *,$	14.67 ± 2.58 *,$
GIIIb	17.83 ± 1.47 *,$	16.33 ± 3.14 *,$
GIVa	32.50 ± 2.59 *,$,#,^	29.50 ± 1.05 *,$,#,^
GIVb	41.83 ± 5.42 *,$,#,^,†	35.17 ± 5.74 *,$,#,^,†
η^2^ (Eta-squared)	0.988	0.991

Data are expressed as mean ± SD (*n* = 6). Significance = *p* < 0.05; * significant vs. GI; $ significant vs. GII; # significant vs. GIIIa; ^ significant vs. GIIIb; † significant vs. GIVa.

**Table 4:** Number of errors during probe trials (PT1 & PT2) in the experimental groups.

Group	PT1	PT2
GI	3.17 ± 1.17	2.17 ± 0.75
GII	12.33 ± 0.52 *	11.17 ± 0.75 *
GIIIa	1.17 ± 0.41 *,$	0.33 ± 0.52 *,$
GIIIb	1.17 ± 0.41 *,$	0.50 ± 0.55 *,$
GIVa	4.50 ± 0.84 *,$,#,^	3.33 ± 0.52 *,$,#,^
GIVb	4.50 ± 0.83 *,$,#,^,†	4.17 ± 1.17 *,$,#,^,†
η^2^ (Eta-squared)	0.982	0.989

Data are expressed as mean ± SD (*n* = 6). Significance = *p* < 0.05; * significant vs. GI; $ significant vs. GII; # significant vs. GIIIa; ^ significant vs. GIIIb; † significant vs. GIVa.

**Table 5:** Modified T-maze performance (% of correct choices) and rotarod latency to fall (sec) in the experimental groups.

Group	Modified T-Maze (%)	Rotarod Latency (Sec)
GI	65.0 ± 5.48	46.5 ± 3.78
GII	23.33 ± 5.16 *	35.0 ± 4.05 *
GIIIa	81.67 ± 7.53 *,$	59.33 ± 0.82 *,$
GIIIb	78.33 ± 4.08 *,$	58.67 ± 1.97 *,$
GIVa	48.33 ± 7.53 *,$,#,^	48.17 ± 3.89 $,#,^
GIVb	40.0 ± 8.94 *,$,#,^,†	45.83 ± 3.66 $,#,^,†
η^2^ (Eta-squared)	0.968	0.884

Data are expressed as mean ± SD (*n* = 6). Significance = *p* < 0.05; * significant vs. GI; $ significant vs. GII; # significant vs. GIIIa; ^ significant vs. GIIIb; † significant vs. GIVa.

**Table 6:** Homogenate levels of hippocampal BDNF, and serum levels of MDA, and SOD.

Group	BDNF (ng/mL)	MDA (nmol/mL)	SOD (U/mL)
GI	1.53 ± 0.38	1.96 ± 0.31	42.47 ± 4.04
GII	0.69 ± 0.22 *	7.09 ± 0.36 *	28.89 ± 6.73 *
GIIIa	6.50 ± 0.21 *,$	2.98 ± 0.34 *,$	159.53 ± 6.26 *,$
GIIIb	6.41 ± 0.13 *,$,#	2.96 ± 0.46 *,$,#	138.89 ± 6.54 *,$,#
GIVa	4.27 ± 0.18 *,$,#,^	4.26 ± 0.25 *,$,#,^	108.70 ± 5.41 *,$,#,^
GIVb	4.16 ± 0.12 *,$,#,^,†	4.88 ± 0.34 *,$,#,^,†	93.11 ± 6.26 *,$,#,^,†
η^2^ (Eta-squared)	0.997	0.992	0.998

Data are expressed as mean ± SD (*n* = 6). Significance = *p* < 0.05; * significant vs. GI; $ significant vs. GII; # significant vs. GIIIa; ^ significant vs. GIIIb; † significant vs. GIVa.

**Table 7:** Granule Cell Layer (GCL) thickness (µm) and number of immunostained neurons/10 HPF.

Group	GCL Thickness	Number of Immunostained Neurons
GI	69.1 ± 22.6	25.4 ± 3.5
GII	26.6 ± 6.7 *	7.8 ± 1.6 *
GIIIa	74.1 ± 14.4 $	48.2 ± 7.3 *,$
GIIIb	69.9 ± 14.6 $	31.1 ± 6.4 $,#
GIVa	54.0 ± 15.1 *,$,#,^	25.7 ± 6.7 $
GIVb	48.4 ± 10.4 *,$,#,^,†	15.8 ± 5.7 *,$,#,^,†

Data are expressed as mean ± SD (*n* = 6). Significance = *p* < 0.05; * significant vs. GI; $ significant vs. GII; # significant vs. GIIIa; ^ significant vs. GIIIb; † significant vs. GIVa.

## Data Availability

Data will be available from the corresponding author. The authors used ChatGPT (OpenAI, San Francisco, CA, USA; version 5.5, 2025) as an AI-assisted writing tool to prepare the graphical abstract. The authors carefully reviewed and edited the modified content and take full responsibility for the final content and integrity of the graphical abstract.
